# Comparison of dental anesthetic efficacy between the periodontal intraligamentary anesthesia and other infiltration anesthesia: a systematic review and meta-analysis

**DOI:** 10.7717/peerj.15734

**Published:** 2023-07-24

**Authors:** Jialei Pan, Yan Wang, Yuran Qian, Jing Zou, Qiong Zhang

**Affiliations:** 1 Sichuan University, State Key Laboratory of Oral Diseases, National Clinical Research Center for Oral Diseases, West China Hospital of Stomatology, Chengdu, China; 2 Sichuan University, State Key Laboratory of Oral Diseases, National Clinical Research Center for Oral Diseases & Department of Pediatric Dentistry, West China Hospital of Stomatology, Chengdu, China; 3 Sichuan University, State Key Laboratory of Oral Diseases, National Clinical Research Center for Oral Diseases & Department of Orthodontics, West China Hospital of Stomatology, Chengdu, China

**Keywords:** Dental local anesthesia, Intraligamentary injection, Infiltration anesthesia

## Abstract

**Background:**

Uncertainty exists regarding the pain scores and the success rate of intraligamentary anesthesia compared to other infiltration anesthesia. Based on the conditions of clinical anesthesia techniques, we conducted a systematic review and meta-analysis to compare the efficacy of intraligamentary anesthesia with other infiltration anesthesia.

**Methods:**

The search was carried out in PubMed Central, Cochrane Central Register of Controlled Trials, MEDLINE (via OVID), Embase (via OVID), and Scopus from the inception to March 26, 2023.

**Results:**

Seven eligible randomized controlled trials were included in the meta-analysis. The results indicated no significant difference in the success rate (RR = 0.96; 95% CI [0.81–1.14]; *p* = 0.65; I^2^= 73%) and visual analog scale (VAS) during dental procedures (MD = 3.81; 95% CI [−0.54–8.16]; *p* = 0.09; I^2^= 97%) between intraligamentary anesthesia and other infiltration anesthesia. However, intraligamentary anesthesia exhibited a higher VAS score during injection than other infiltration anesthesia (MD = 8.83; 95% CI [4.86–12.79]; *p* < 0.0001; I^2^= 90%). A subgroup analysis according to infiltration techniques showed that supraperiosteal anesthesia had a lower VAS score during dental procedures than intraligamentary anesthesia.

**Conclusions:**

Intraligamentary anesthesia and other infiltration anesthesias have the same success rate and pain during dental procedures. However, the pain during injection of intraligamentary anesthesia is heavier than that of other infiltration anesthesia.

## Introduction

Efficient pain management is essential for a successful dental procedure. Dental local anesthesia, including nerve blocks and various infiltration techniques, is widely used to block pain (Badr & Aps, 2018). However, anesthesia itself has become one of the main factors preventing patients from accepting dental treatment (Milgrom et al., 1997). The type of anesthesia technique affects pain levels during injections and treatment (Kaufman et al., 2005).

Subperiosteal infiltration along with inferior alveolar nerve block (IANB) are among the most commonly used anesthesia for endodontic treatment and exodontia of mandibular molars. However, subperiosteal anesthesia is associated with various complications, and it is difficult to confirm that the needle has reached the subperiosteum. Supraperiosteal anesthesia, a local infiltration anesthesia, is routinely employed in the maxilla. The injection site is shallow, indicating the pain scores during injection a bit lower. It can reduce the rate of nerve injury and local infection caused by the injection, making it a promising technique. However, it leads to unsteady success rates and pain scores in maxillary teeth (Haas et al., 1991; Kennedy et al., 2001).

Another infiltration anesthesia, *i.e.,* intraligamentary anesthesia, is suggested as an alternative, in which the anesthetic agent is injected into the periodontal ligament between the tooth and the alveolar bone. It is a preferred primary technique for single-tooth anesthesia with a limited soft tissue without causing lip and facial muscle(s) numbness (Meechan, 1992). Intraligamentary anesthesia can also be used as a supplementary anesthesia when the effect of block anesthesia is not satisfactory. The success rate of intraligamentary anesthesia for dental procedures is as high as 98.4% (Miller, 1983). It is suitable for hemophiliac patients due to no complications regarding hemorrhage and hematoma formation (Ah Pin, 1987; Spuller, 1988). Rare cases of adverse responses have been reported with this technique (Malamed, 1982).

Similar to intraligamentary anesthesia, intraseptal anesthesia only works on limited skin and individual teeth. Although the contraindications include acute inflammation or infection at the injection site, intraseptal anesthesia remains a convenient local anesthesia for general dental surgeons (Woodmansey, 2005). This technique has instantaneous onset of action but it may require extensive clinical training, and the period of efficacy is short-lived. Intraseptal anesthesia can overcome inconveniences associated with the intraligamentary anesthesia when used under the guidelines recommended.

To date, the comparison of pain scores and success rates between intraligamentary anesthesia and other infiltration anesthesia remains controversial. Some literature showed no significant difference between these techniques in pain scores (Ram & Peretz, 2003; Fan et al., 2009), with some indicating either higher or lower pain scores during the administration of intraligamentary anesthesia injection than other local infiltration techniques (Mansour & Adawy, 1985; Marin, 1987; Kaufman et al., 2005). This meta-analysis and systematic review aimed to compare the efficacy of intraligamentary anesthesia and other infiltration anesthesias by focusing on several clinical parameters, the success rate of anesthesia, pain during injection and dental procedures.

## Materials and Methods

This review was registered in Protocol registration: PROSPERO 2021 CRD42021234105. The study was performed according to Preferred Reporting Items for Systematic Reviews and Meta-Analyses (PRISMA) (Shamseer et al., 2015) and the Cochrane Handbook for Systematic Reviews of Interventions (Higgins et al., 2022).

### Literature search

We searched PubMed Central, Cochrane Central Register of Controlled Trials, MEDLINE (*via* OVID), Embase (*via* OVID), and Scopus from the inception to March 26, 2023. The following combinations were used to identify studies: (“anesthesia” or “injection”) and (“intraligamental” or “intraligamentary” or “periodontal ligament”) and (“subperiosteal” or “intraseptal” or “supraperiosteal” or “infiltration”). The search strategies were shown in Table 1.

**Table 1:** Search strategies for five databases.

**Electronic databases and search strategies**	
PubMed Central	#1 (”Anesthesia”[Mesh]) #2 ((((”Injections”[Mesh]) OR (Injectable)) OR (Injectables)) OR (Injection)) #3 #1 OR #2 #4 ((”Periodontal Ligament”[Mesh]) OR (intraligamentary)) OR (intraligamental) #5 (((subperiosteal) OR (intraseptal)) OR (supraperiosteal)) OR (infiltration) #6 #3 AND #4 AND #5
Embase	#1 ’anesthesia’/exp OR ’anaesthesia’ OR ’anesthetization’ #2 ’injection’/exp OR ’injections’ #3 #1 OR #2 #4 ’periodontal ligament’/exp OR ’intraligamentary’ OR ’intraligamental’ #5 ’subperiosteal’ OR ’intraseptal’ OR ’supraperiosteal’ OR ’infiltration’ #6 #3 AND #4 AND #5
MEDLINE	1 exp Injections/ 2 exp Anesthesia/ 3 1 or 2 4 exp Periodontal Ligament/ 5 intraligamental.mp. 6 intraligamentary.mp. 7 4 or 5 or 6 8 subperiosteal.mp. 9 intraseptal.mp. 10 supraperiosteal.mp. 11 infiltration.mp. 12 8 or 9 or 10 or 11 13 3 and 7 and 12
Cochrane Library	#1 MeSH descriptor: [Anesthesia] explode all trees #2 MeSH descriptor: [Injections] explode all trees #3 (Injection) OR (Injectables) OR (Injectable) #4 #1 OR #2 OR #3 #5 MeSH descriptor: [Periodontal Ligament] explode all trees #6 (intraligamental) OR (intraligamentary) #7 #5 OR #6 #8 (subperiosteal) OR (intraseptal) OR (supraperiosteal) OR (infiltration) #9 #4 AND #7 AND #8
Scopus	( ALL ( ( subperiosteal OR intraseptal OR supraperiosteal OR infiltration ) ) AND ALL ( ( injection OR anesthesia ) ) AND ALL ( ( intraligamental OR intraligamentary OR {periodontal ligament} ) ) )

### Inclusion criteria

The inclusion criteria for this study were as follows: (1) randomized controlled trials (RCTs); (2) comparing intraligamentary anesthesia with another oral infiltration anesthetic technique during dental procedures; and (3) studies published in English.

### Exclusion criteria

The studies were excluded based on the following criteria: (1) if the studies were reviews, systematic reviews, viewpoints, perspectives, or correspondences; (2) if the studies were non-human experiments; and (3) if the study did not report the necessary data.

### Study selection, data extraction, and quality assessment

We used EndNote X9 to exclude data duplication after importing all the literature search histories in this software. Then, two reviewers (YZ and JW) independently read all the titles and abstracts of publications retrieved through our search. We obtained any papers considered suitable for the review in their full texts, including those for which a decision could not be made from just the title and abstract. In cases of disagreement, a third person was involved (QZ).

After completing the literature search, two reviewers (JP and YW) extracted the data of the included studies independently using a predefined data extraction form. Disagreements were resolved through discussion or consultation with a third reviewer (QZ).

The quality of included studies was also assessed independently by two reviewers (JP and YW). The assessment was based on the Cochrane Handbook for Systematic Reviews of Interventions (Higgins et al., 2022).

### Data analysis

Review-Manager software (RevMan, Version 5.4.1 Windows; The Cochrane Collaboration, Oxford, UK) was used for the meta-analysis. Continuous outcomes, such as the visual analog scale (VAS) during injection and VAS during the dental procedure, were expressed as the mean difference (MD) with the respective 95% CIs. The binary outcome (the success rate of anesthesia) was expressed as the risk ratio (RR) with 95% CIs. Statistical significance was set at *p* < 0.05. Heterogeneity in the forest plot was evaluated using the Cochrane chi-square-based Q-test and regarded as significant if the *p*-value was less than 0.1. Meanwhile, the I^2^ statistic was used to test for heterogeneity efficiently, where I^2^ < 25%, I^2^ = 25–50%, and I^2^ > 50% indicated low, moderate, and high degrees of heterogeneity, respectively. Sensitivity analysis was conducted to identify potential sources of heterogeneity by removing the included study one after another. A random-effects model was adopted when there was statistical evidence of heterogeneity. A subgroup analysis was conducted to identify whether the type of infiltration anesthesia, sample size, and adrenaline concentration affected the VAS during dental procedures and success rate. Begg’s funnel plot and Egger’s linear regression tests (Egger et al., 1997) were conducted.

## Results

### Search results

**Figure 1:**
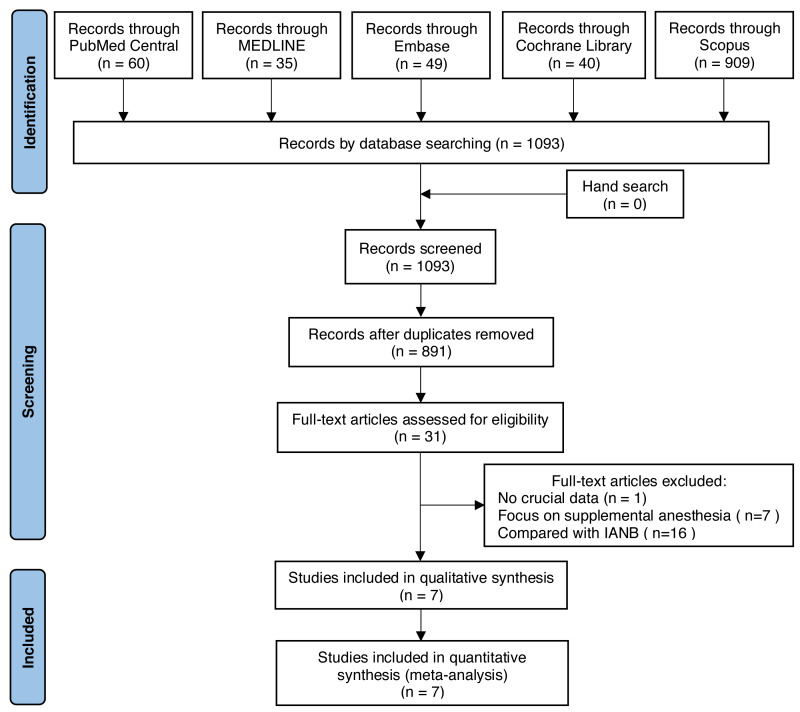
Flow chart of the reviewing process.

A total of 1,093 records were identified from five databases. After removing the duplicates, 891 potentially relevant abstracts were initially screened, and 860 were excluded according to the inclusion criteria. We retrieved and reviewed 31 full-text articles. Finally, only seven studies (Meechan & Ledvinka, 2002; Brkovic et al., 2010; Biocanin et al., 2013; Al-Shayyab, 2017; Jain & Nazar, 2018; Ryalat et al., 2018; Subramaniam, Dhanraj & Jain, 2018) involving 386 subjects were included in the meta-analysis (Fig. 1).

### Characteristics and quality of included studies

Table 2 summarizes the general features of seven eligible studies. These studies were published between 2002 and 2018 and were conducted in Serbia, India, Jordan, and the UK. Of seven RCTs, four used a split-mouth design, and three used a parallel design. The split-mouth design divides the patients’ dentition into halves and randomly assign two different treatments to one side. Each patient can act as their own control group. While the parallel design assigns patients to intervention and control groups randomly. The experimental groups of four studies were given supraperiosteal anesthesia, two were given intraseptal anesthesia, and one was given subperiosteal infiltration. All the studies had performed intraligamentary anesthesia as a control. As for split-mouth trials, studies with intraligamentary anesthesia were set as control groups and those with other intraseptal anesthesia were set as experimental groups. Of all the studies, six studies provided data on VAS during the dental procedure, three provided data on VAS during injection, and two provided the verbal rating scale. The VAS is a tool for statistically measuring pain, graded from 0 (no pain) to 100 (pain as bad as it could be) (Jensen, Karoly & Braver, 1986). Patients, instructed before the study, provide a score from 0 to 100 on a 100-mm scale according to their pain. The verbal rating scale has a variable number of gradually ascending verbal descriptors. With the verbal rating scale, patients rate their feelings according to the descriptors they are given.

**Figure 2:**
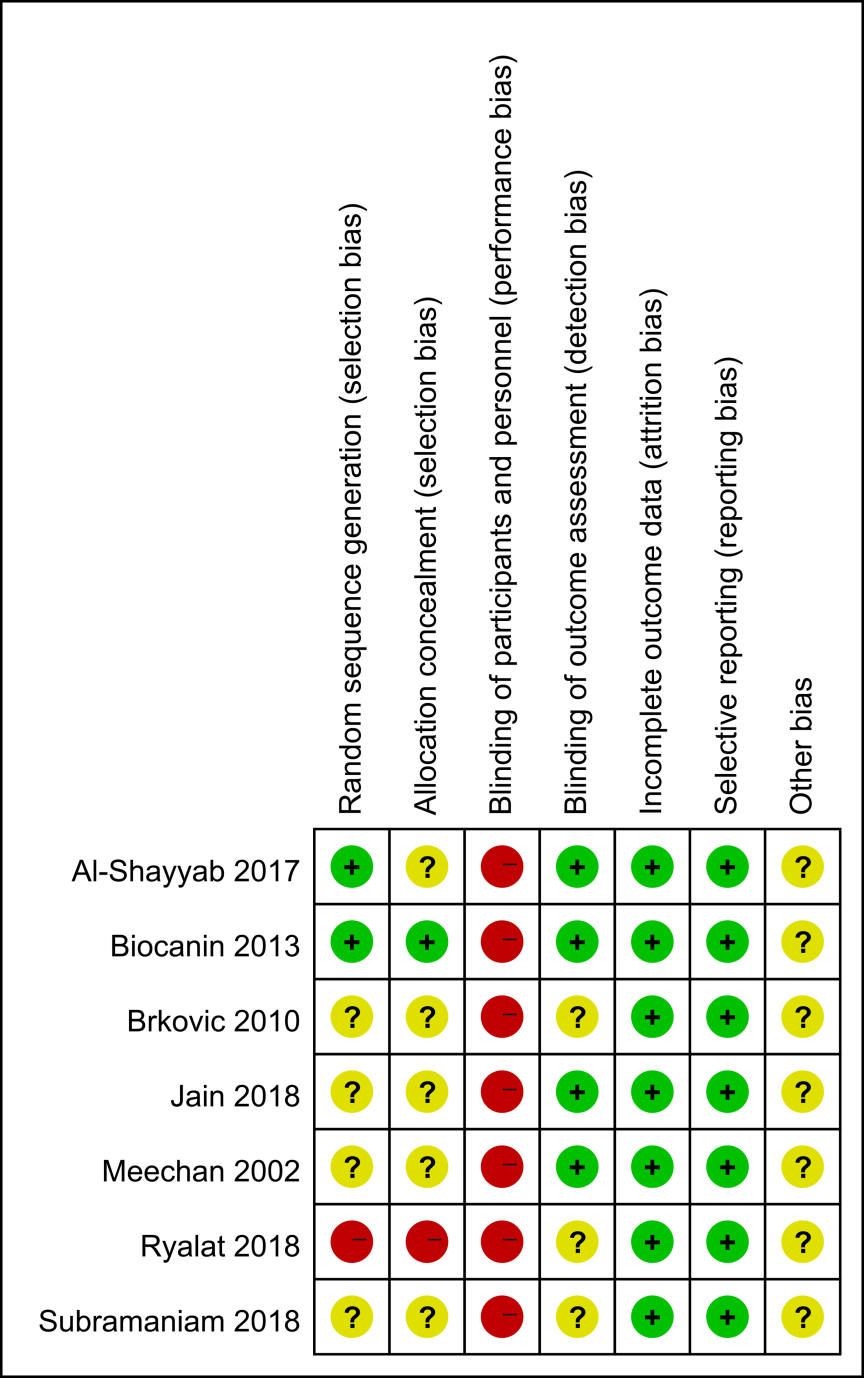
Risk of bias of the included RCTs: review authors’ judgments about each risk of bias item for each included study. Symbols: +, low risk of bias; -, high risk of bias; ?, unclear risk of bias.

Figure 2 presents the quality assessment. Four studies (Meechan & Ledvinka, 2002; Brkovic et al., 2010; Jain & Nazar, 2018; Subramaniam, Dhanraj & Jain, 2018) did not report how they produced random sequences. Thus, their biases of random sequence generation were judged as unclear. One study (Ryalat et al., 2018) used alternate allocation, indicating that its bias of allocation concealment was high. Since the surgeon had to perform the procedures, a high risk of performance bias might exist in every study. Moreover, several domains were judged as unclear in this study because of the lack of information.

### VAS during injection

**Figure 3:**
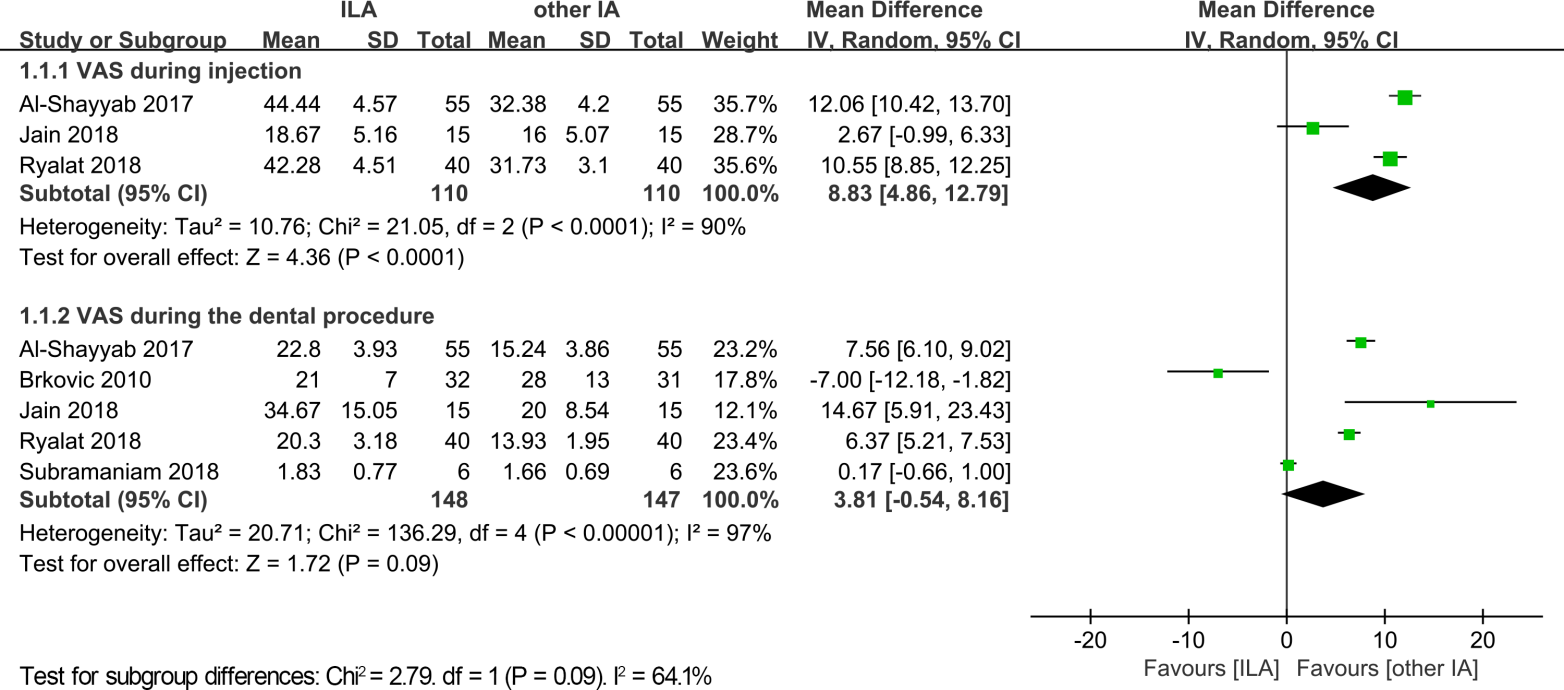
Forest plot of VAS during injection and VAS during the dental procedure. SD, standard deviation; CI, confidence interval; VAS, visual analog scale.

Three trials with 220 cases reported VAS scores during injection for both the intraligamentary anesthesia and other infiltration anesthesia groups. The pooled results indicated that the other infiltration anesthesia group had a lower VAS score (MD = 8.83; 95% CI [4.86–12.79]; *p* < 0.0001, Fig. 3) with a high heterogeneity (*p* < 0.0001, I^2^ = 90%).

**Table 2:** The general characteristics of the included studies.

**Study, Year**	**Country**	**Study** **type**	**Age** **(years)**	**Gender** **(female/ male)**	**Anesthesia** ** technique** **(C/I)**	**Number** **of ** **patients** **(C/I)**	**Number of** ** Successful** ** anesthesia** **(C/I)**	**Anesthetized tooth** ** position**	**Outcomes**
Al-Shayyab (2017)	Jordan	RCT (split mouth)	mean ±SD: 34.87 ± 14.93; range:13–65	23/32	intraligamentary anesthesia/ supraperiosteal anesthesia	55/55	39/49	Posterior maxillary permanent teeth	VAS, success rate, VRS
Biocanin et al. (2013)	Serbia	RCT (parallel)	mean ±SD: 27.8 ± 9.9; range: 24–31	28/32	intraligamentary anesthesia/ intraseptal anesthesia	30/30	21/27	Mandibular first premolars	success rate, onset time, duration of anesthesia, the width of the anesthetic field
Brkovic et al. (2010)	Serbia	RCT (split mouth)	mean ±SD: 37.1 ± 12.3	19/16	intraligamentary anesthesia/ intraseptal anesthesia	35/35	32/31	Maxillary lateral incisors extraction	VAS, success rate, onset time, duration of anesthesia
Jain & Nazar (2018)	India	RCT (parallel)	range:18–40	NR	intraligamentary anesthesia/ supraperiosteal anesthesia	15/15	15/15	Maxillary teeth	VAS, success rate
Meechan & Ledvinka (2002)	UK	RCT (split mouth)	range: 21–24	NR	intraligamentary anesthesia/ supraperiosteal anesthesia	12/12	0/10	Mandibular central incisors	VAS, success rate
Ryalat et al. (2018)	Jordan	RCT (split mouth)	mean: 32.15; range: 11–65	11/29	intraligamentary anesthesia/ supraperiosteal anesthesia	40/40	34/25	Maxillary first molar teeth	VAS, VRS, success rate
Subramaniam, Dhanraj & Jain (2018)	India	RCT (parallel)	range: 25–40	4/8	intraligamentary anesthesia/ subperiosteal infiltration	6/6	6/6	NR	VAS, success rate

**Notes.**

SD standard deviation C control group I intervention group NR not reported RCT randomized controlled trial VAS visual analog scale

### VAS during the dental procedure

VAS during the dental procedure was recorded in five trials, with 148 cases in the intraligamentary anesthesia group and 147 cases in the other infiltration anesthesia group. There was no significant difference between the intraligamentary anesthesia and other infiltration anesthesia groups with a high heterogeneity (MD = 3.81; 95% CI [−0.54–8.16]; *p* = 0.09; I^2^ = 97%, Fig. 3).

### Success rate

**Figure 4:**
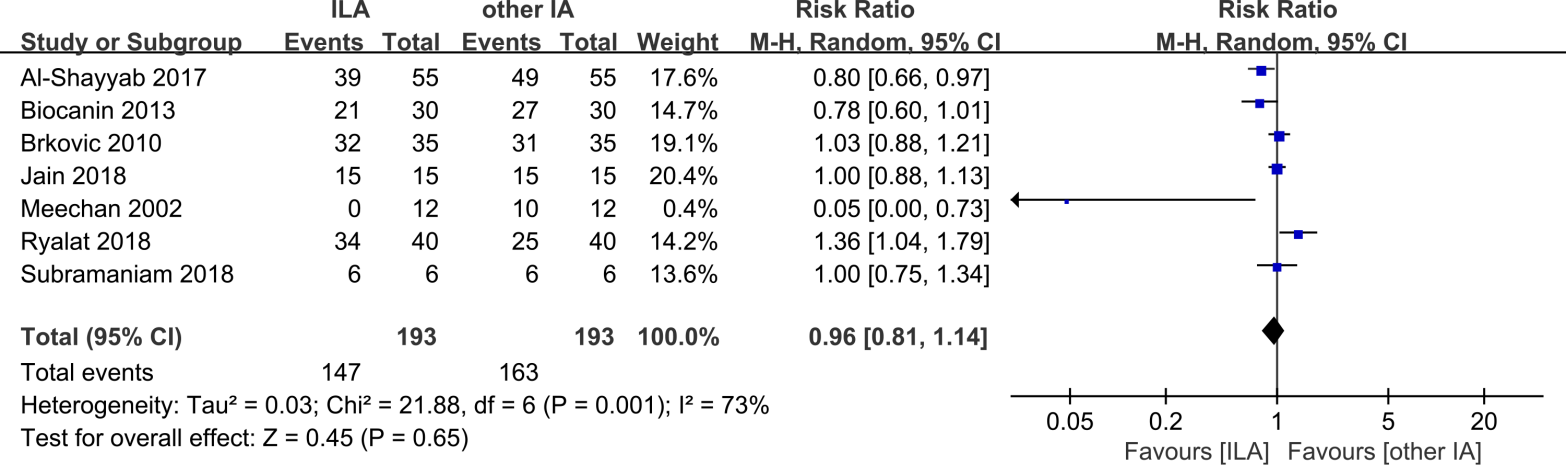
Forest plot of success rate of the two groups. CI, confidence interval.

An adequate depth of anesthesia after the first injection was defined as success. The anesthesia would be considered failed if insufficient anesthesia was produced and further injections were required. Seven included studies referred to success rates of both intraligamentary anesthesia and other infiltration anesthesia techniques. Only two of the seven studies applying intraligamentary anesthesia showed a higher success rate than other infiltration anesthesia techniques. The other studies reached a consensus that intraligamentary anesthesia had a lower success rate with a high heterogeneity (RR = 0.96; 95% CI [0.81–1.14]; *p* = 0.65; I^2^ = 73%, Fig. 4). In total, there was no significant difference between intraligamentary anesthesia and other infiltration anesthesia techniques.

### Sensitivity analysis

Sensitivity analysis was carried out by excluding the studies one by one to examine whether an individual study affected the results. When studies were removed one at a time in analyzing the VAS during the dental procedure and success rate, the statistical I^2^ was not markedly altered by any single study, indicating the robustness of the results (Tables 3 and 4). However, the sensitivity analysis in VAS during the injection illustrated that the study by Jain & Nazar (2018) was the source of high heterogeneity (Table 5).

**Table 3:** Sensitivity analysis of VAS during the dental procedure.

		**Heterogeneity**			**Meta-analysis**	
	**No. of studies**	**I** ^**2**^ **(%)**	**P**	**Model**	**Mean difference**	**95% CI**
Subgroup analysis						
Anesthesia of infiltration anesthesia						
Intraseptal anesthesia	1	NA	NA	NA	−7	−12.18–1.82
Supraperiosteal anesthesia	3	57	0.10	Random-effect	7.21	5.49–8.92
Subperiosteal infiltration	1	NA	NA	NA	0.17	−0.66–1.00
Sample size						
≤30	2	90	0.001	Random-effect	6.74	−7.41–20.88
>30	3	97	<0.00001	Random-effect	3.61	−0.43–7.65
Adrenaline concentration						
1:80000	2	99	<0.00001	Random-effect	3.26	−2.82–9.33
1:100000	2	96	<0.00001	Random-effect	0.50	−13.76–14.76
1:200000	1	NA	NA	NA	14.67	5.91–23.43
Sensitivity analysis						
Brkovic et al., 2010 excluded	4	98	<0.00001	Random-effect	6.10	1.47–10.73
Subramaniam et al., 2018 excluded	4	90	<0.00001	Random-effect	4.95	1.07–8.82
Al-Shayyab et al., 2017 excluded	4	97	<0.00001	Random-effect	2.70	−2.38–7.79
Jain & Nazar, 2018 excluded	4	98	<0.00001	Random-effect	2.33	−2.24–6.90
Ryalat et al., 2018 excluded	4	97	<0.00001	Random-effect	3.16	−2.66–8.99

**Table 4:** Sensitivity analysis of success rate.

		**Heterogeneity**			**Meta-analysis**	
	**No. of studies**	**I** ^**2**^ **(%)**	**P**	**Model**	**Risk ratio**	**95% CI**
Subgroup analysis						
Anesthesia of infiltration anesthesia						
intraseptal anesthesia	2	74	0.05	Random-effect	0.91	0.68–1.23
supraperiosteal anesthesia	4	84	0.0004	Random-effect	0.98	0.71–1.34
subperiosteal infiltration	1	NA	NA	NA	1	0.75–1.34
Sample size						
≤30	2	0	1.00	Random-effect	1.00	0.89–1.12
>30	5	80	0.0006	Random-effect	0.94	0.72–1.22
Adrenaline concentration						
1:80000	3	81	0.006	Random-effect	1.02	0.56–1.84
1:100000	3	69	0.04	Random-effect	0.87	0.71–1.07
1:200000	1	NA	NA	NA	1.36	1.04–1.79
Sensitivity analysis						
Brkovic et al., 2010 excluded	6	77	0.0006	Random-effect	0.94	0.76–1.17
Subramaniam et al., 2018 excluded	6	77	0.0006	Random-effect	0.96	0.79–1.16
Meechan 2002 excluded	6	63	0.02	Random-effect	0.97	0.85–1.11
Al-Shayyab et al., 2017 excluded	6	68	0.009	Random-effect	1.00	0.84–1.19
Jain 2018 excluded	6	75	0.001	Random-effect	0.95	0.76–1.18
Ryalat 2018 excluded	6	79	0.0002	Random-effect	0.90	0.74–1.11
Biocanin 2013 excluded	6	72	0.003	Random-effect	1.00	0.84–1.19

**Table 5:** Sensitivity analysis of VAS during injection.

		**Heterogeneity**			**Meta-analysis**	
	**No. of studies**	**I** ^**2**^ **(%)**	**P**	**Model**	**Mean difference**	**95% CI**
Sensitivity analysis						
Al-Shayyab et al. 2017 excluded	2	93	0.0001	Random-effect	6.78	−0.93–14.50
Jain & Nazar, 2018 excluded	2	36	0.21	Random-effect	11.32	9.84–12.80
Ryalat et al., 2018 excluded	2	95	<0.00001	Random-effect	7.51	−1.68–16.71

### Publication bias

**Figure 5:**
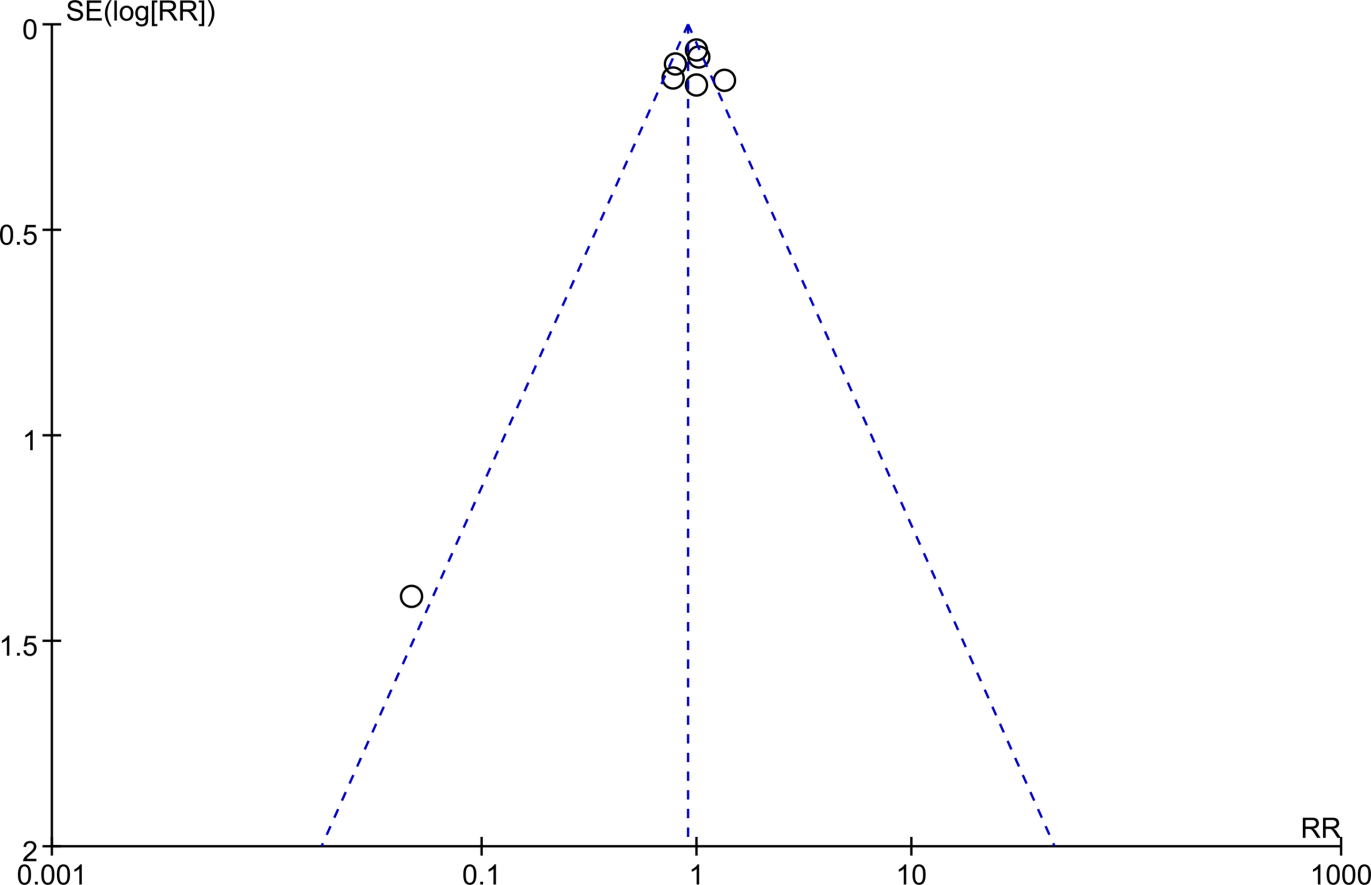
The funnel plot of the studies included in the meta-analysis.

Begg’s funnel plot and Egger’s test were performed to evaluate the publication bias of the included studies. The result of Begg’s funnel plot is shown in Fig. 5. The Egger’s tests were performed for VAS during injection (*p* = 0.147), VAS during the dental procedure (*p* = 0.685), and success rate (*p* = 0.573), respectively. None of the tests indicated significant publication bias.

### Verbal rating scale

Only two of the eligible studies (Al-Shayyab, 2017; Ryalat et al., 2018) recorded the verbal rating scale. Clinicians asked the patients to describe pain during dental procedures as less than expected, as expected, or greater than expected. The differences in the verbal rating scale data for dental procedures were statistically significant between the intraligamentary anesthesia and other infiltration anesthesia groups in both studies. None of the patients in these two trials described the extraction pain as “less than expected” in the intraligamentary anesthesia group. Therefore, there appeared to be a higher proportion of “as expected” and “greater than expected” cases in the intraligamentary anesthesia group than in the other infiltration anesthesia group. Additionally, in Al-Shayyab (2017), the patients were asked to describe the extraction pain as acceptable or unacceptable. It turned out that the proportion of “acceptable” cases was higher in the other infiltration anesthesia group. Compared with VAS, verbal rating scale is less sensitive but more simply ranked. Overall, the outcome is consistent with VAS scores, indicating that supraperiosteal anesthesia is more effective than intraligamentary anesthesia.

### Adverse events

One of the included studies (Biocanin et al., 2013) reported adverse events of anesthesia. Of 90 patients receiving the intraseptal anesthesia, three reported a slight hematoma of the papilla region. In addition, the sensitivity of tooth to biting was recorded in five out of 90 patients receiving the intraligamentary anesthesia. The difference in local side effects between the two techniques might be attributed to their different injection sites.

## Discussion

This systematic review and meta-analysis included seven studies, with a total of 386 patients. Only RCTs were included. The overall risk of bias in all the included studies was high based on the Cochrane risk-of-bias tool. The summary of the results showed that intraligamentary anesthesia caused more pain than supraperiosteal anesthesia during the injection with high heterogeneity. A subgroup analysis was carried out for VAS during dental procedures to compare the pain scores of intraligamentary anesthesia and every specific infiltration anesthesia. The results showed lower pain scores of intraligamentary anesthesia than intraseptal anesthesia but higher than that of supraperiosteal anesthesia. There was no difference in pain scores between intraligamentary anesthesia and subperiosteal infiltration during dental procedures. Concerning success rates, the results also showed no significant difference between intraseptal anesthesia and other infiltration anesthesia techniques.

As the heterogeneity was extremely high, a sensitivity analysis was carried out to determine their impact. The analysis did not find any single study influencing the results in both VAS during dental procedures and success rate, indicating the robustness of the outcomes. For VAS during injection, the statistical I^2^ dropped significantly from 90% to just 36% after removing Jain & Nazar’s (2018) trial. Reading and comparing the three articles showed that the VAS scores in Jain & Nazar’s (2018) study were only half of those in the other two articles. The validity and reliability of VAS are affected by several factors, such as learning, memory, and perceptual judgment (Carlsson, 1983). Besides, patients have to accept pre-training to be able to use VAS before the procedure and understand the concept of the method and the relationship between measured pain and real pain. However, doctors themselves exhibit differences in comprehending the corresponding relationship, resulting in significant differences between different experiments. We also carried out a subgroup analysis based on the infiltration anesthesia technique, adrenaline concentration, and sample size to explore the possible causes of heterogeneity. However, we could not rule out the possibility that these factors caused heterogeneity. In addition, we retrieved an RCT comparing the effects of intraligamentary anesthesia and supraperiosteal anesthesia in children (Elbay et al., 2016). The results showed that pain during needle insertion was greater with intraligamentary anesthesia anesthesia than with supraperiosteal anesthesia in children, consistent with our conclusion in adults.

Several measurement tools have been developed to evaluate the efficacy of anesthesia (Breivik et al., 2008). A 100-mm VAS is the most frequently used tool for measuring pain intensity (Williams, Davies & Chadury, 2000; Lara-Muñoz et al., 2004; Hjermstad et al., 2011). The VAS does not require verbal or reading skills and can be employed in various settings (Younger, McCue & Mackey, 2009). However, it needs a clear vision, dexterity, pen and paper, or an electronic display and cannot be used remotely (Williamson & Hoggart, 2005). Nevertheless, these disadvantages do not affect dental anesthesia. As a continuous scale, the VAS has high sensitivity. Apart from judging whether the patient can feel pain, the VAS characterizes the degree of pain that the patient feels. However, the VAS has an inevitable defect: it is highly subjective. The results rely on individuals’ ability to convert pain intensity to an abstract scale (Williams, Davies & Chadury, 2000; Reed & Van Nostran, 2014), resulting in heterogeneities among different studies. The verbal rating scale is a categorical, ordinal scale, which can also effectively assess subjective pain. It is easy and convenient for clinicians to use the verbal rating scale. Patients can select one of the pain descriptors without any pre-training, and the clinicians can directly estimate patients’ general distress levels. However, its use is limited by patients’ vocabulary and abilities of expression.

The verbal rating scale also lacks the sensitivity of the VAS in detecting small changes in pain intensity (Jensen, Karoly & Braver, 1986). Both the VAS and verbal rating scale are valid, consistent, and reproducible (Lara-Muñoz et al., 2004). In addition, to measure the unpleasantness or affective dimension of children, the old, and the disabled, other tools can be selected. These individuals have a poor ability to rate their pain. Tools like Wong-Baker Faces Pain Rating Scale (Wong & Baker, 1988) and standardized color analog scale can help patients express their feelings through colors and child faces. However, these tools have poor responsiveness (O’Rourke, 2004). Overall, clinicians need to select a suitable measuring tool according to the conditions. Notably, previous studies suggested that conclusions from different scales should not be used interchangeably in the clinical setting or for increased statistical power (Bailey et al., 2007; Kliger et al., 2015).

Various factors can influence the practical effects of anesthesia. Personal traits, including gender, age, experience, systemic conditions, and genetic factors, also impact an individual’s feeling of pain (Crowley, 2007; Moore, Straube & Aldington, 2013; Ehde, Dillworth & Turner, 2014). Besides, local circumstances of dental tissues impact the efficacy, too (Nusstein, Reader & Drum, 2010). Pain can be reduced by keeping anesthetic agents at a temperature similar to body temperature and slowing the injection speed (Malamed, 1998). The use of different anesthetics and anesthesia techniques can also significantly affect the efficacy of pain control (St George et al., 2018).

For clinical use, clinicians have to be aware of the characteristics of different anesthesia and combine them with patient’s conditions for selecting suitable anesthesia. IANB and supraperiosteal anesthesia are the most commonly used local anesthesia. However, both have the disadvantage of a long-lasting effect, which is hard to control (Foster et al., 2007; Lasemi et al., 2015). IANB also has complications that include nerve injury, accidental intravascular injection, and hematoma (Wright, 2011). Intraligamentary anesthesia has a shorter and controllable operation time. Besides, intraligamentary anesthesia only anesthetizes specific teeth instead of anesthetizing all the teeth in the quadrant and soft tissues like the lip and the tongue. The benefits of intraligamentary anesthesia also include avoiding nerve injuries and accidental intravascular injection, which can reduce the risk of cardiovascular disturbances (Lilienthal, 1975; Liau et al., 2008).

According to our results and previous research, the success rate of intraligamentary anesthesia is not significantly different from infiltration anesthesia or IANB (Shabazfar et al., 2014). Moreover, concerning injection pain, the VAS scores of infiltration anesthesia are lower than intraligamentary anesthesia, and the scores of intraligamentary anesthesia are lower than IANB. Intraligamentary anesthesia can be an effective alternative to IANB since it avoids the potential complications associated with IANB (Youssef et al., 2021; Yılmaz & Çağırır Dindaroğlu, 2023).

There are various approaches to painless treatment. For dental treatments, supplemental injections such as intraligamentary anesthesia, intraosseous anesthesia and infiltration anesthesia are recommended. Supplemental intraligamentary injections following the failure of IANB have been shown to be effective (Gupta et al., 2022). Computer-controlled anesthesia offers certain advantages compared to traditional anesthesia. Computer-controlled intraligamentary anesthesia (CC-ILA) has a higher level of safety and a faster onset *versus* conventional intraligamentary anesthesia. Additionally, CC-ILA is less painful while remaining as effective as IANB (Helmy, Zeitoun & El-Habashy, 2022). When computer-controlled delivery systems were used, patients reported less pain during injections and clinical procedures with supraperiosteal anesthesia as compared to intraligamentary anesthesia, although the rate of postoperative complications was higher for supraperiosteal anesthesia (Elbay et al., 2016).

According to intraligamentary anesthesia characteristics, this anesthesia can be used in the following conditions: routine tooth removal and tooth preparation, supplementary injections after the failure of other anesthesia, and dental procedures for patients with bleeding disorders. Furthermore, the value of intraligamentary anesthesia in the field of oral implantology has been explored (Dalla Torre & Burtscher, 2020); however, more evidence is necessary for its clinical application. In addition, intraligamentary anesthesia’s low risk of side effects indicates its value for use in children. However, higher injection pain might result in less cooperative patients. Thus, the comprehensive benefits of using intraligamentary anesthesia for children are still unclear.

There were several limitations in the present review. This meta-analysis presented an extremely high heterogeneity among the studies included. Before the analysis, we considered that the results would be affected by confounding factors such as infiltration anesthesia, sample size, and adrenaline concentration. However, the sensitivity analysis and subgroup revealed that none of the confounders mentioned above completely explained the observed heterogeneity. Moreover, only seven RCTs were included, and the sample sizes in each trial were not large, possibly affecting the final results. We believe more RCTs on oral local anesthesia are necessary and valuable. Thirdly, the included studies had selection bias and performance bias risks. Some studies (Meechan & Ledvinka, 2002; Brkovic et al., 2010; Jain & Nazar, 2018; Subramaniam, Dhanraj & Jain, 2018) did not report the details of how the patients were randomized. Clinicians have to perform the anesthesia procedures, which means blinding them is impossible. Four, VAS is a subjective index, which is difficult to standardize and easy to impact. Therefore, using VAS as an evaluation index might contribute to the heterogeneity significantly. Because the inclusion criteria include articles should be published in English, there may be studies in other languages that were not included in the analysis.

This meta-analysis of the present published studies revealed that intraligamentary anesthesia was as effective as other infiltration anesthesia. However, intraligamentary anesthesia induces more pain during the injection process than other infiltration anesthesia techniques. Clinically, a computer-controlled anesthetic device can reduce pain during the injection of intraligamentary anesthesia technique (Baghlaf, Elashiry & Alamoudi, 2018). Combined with other characteristics of intraligamentary anesthesia, it should be promoted as a reliable anesthesia for supplementary anesthesia and an alternative for supraperiosteal anesthesia. However, due to the heterogeneity and limitations in the present study, the meta-analysis results might need to be estimated. Further investigations are necessary to confirm our results.

Several operations can be used to improve the quality and efficiency of future research. Concerning random sequence generation, some studies only reported they ‘selected randomly,’ which is unconvincing according to the Cochrane handbook. Besides, important useful data were missing. For further studies, researchers are advised to collect more information, including the onset of anesthesia, duration of anesthesia, duration of the procedure, the width of the anesthetic field, verbal rating scale, and adverse effects. Such information is helpful to guide clinical work and to significantly improve the quality of experiments.

##  Supplemental Information

10.7717/peerj.15734/supp-1 Supplemental Information 1 PRISMA checklist

10.7717/peerj.15734/supp-2 Supplemental Information 2 Systematic Review and Meta-Analysis Rationale

10.7717/peerj.15734/supp-3 Supplemental Information 3 Excluded studies with reasons

## Additional Information and Declarations

### Data Availability

The following information was supplied regarding data availability: This is a literature review and dose not have raw data.

## References

[ref-1] Ah Pin PJ (1987). The use of intraligamental injections in haemophiliacs. British Dental Journal.

[ref-2] Al-Shayyab MH (2017). Periodontal ligament injection versus routine local infiltration for nonsurgical single posterior maxillary permanent tooth extraction: comparative double-blinded randomized clinical study. Therapeutics and Clinical Risk Management.

[ref-3] Badr N, Aps J (2018). Efficacy of dental local anesthetics: a review. Journal of Dental Anesthesia and Pain Medicine.

[ref-4] Baghlaf K, Elashiry E, Alamoudi N (2018). Computerized intraligamental anesthesia in children: a review of clinical considerations. Journal of Dental Anesthesia and Pain Medicine.

[ref-5] Bailey B, Bergeron S, Gravel J, Daoust R (2007). Comparison of four pain scales in children with acute abdominal pain in a pediatric emergency department. Annals of Emergency Medicine.

[ref-6] Biocanin V, Brkovic B, Milicic B, Stojic D (2013). Efficacy and safety of intraseptal and periodontal ligament anesthesia achieved by computer-controlled articaine + epinephrine delivery: a dose-finding study. Clinical Oral Investigations.

[ref-7] Breivik H, Borchgrevink PC, Allen SM, Rosseland LA, Romundstad L, Hals EK, Kvarstein G, Stubhaug A (2008). Assessment of pain. British Journal of Anaesthesia.

[ref-8] Brkovic BM, Savic M, Andric M, Jurisic M, Todorovic L (2010). Intraseptal vs. periodontal ligament anaesthesia for maxillary tooth extraction: quality of local anaesthesia and haemodynamic response. Clinical Oral Investigations.

[ref-9] Carlsson AM (1983). Assessment of chronic pain. I. Aspects of the reliability and validity of the visual analogue scale. Pain.

[ref-10] Crowley KE (2007). Anesthetic issues and anxiety management in the female oral and maxillofacial surgery patient. Oral and Maxillofacial Surgery Clinics of North America.

[ref-11] Dalla Torre D, Burtscher D (2020). Intraligamentary anaesthesia as a possible anaesthetic option in oral implantology: a retrospective analysis. International Journal of Oral and Maxillofacial Surgery.

[ref-12] Egger M, Davey Smith G, Schneider M, Minder C (1997). Bias in meta-analysis detected by a simple, graphical test. BMJ.

[ref-13] Ehde DM, Dillworth TM, Turner JA (2014). Cognitive-behavioral therapy for individuals with chronic pain: efficacy, innovations, and directions for research. The American Psychologist.

[ref-14] Elbay Ü, Elbay M, Kaya E, Cilasun Ü (2016). Intraligamentary and supraperiosteal anesthesia efficacy using a computer controlled delivery system in mandibular molars. The Journal of Clinical Pediatric Dentistry.

[ref-15] Fan S, Chen WL, Pan CB, Huang ZQ, Xian MQ, Yang ZH, Dias-Ribeiro E, Liang YC, Jiao JY, Ye YS, Wen TY (2009). Anesthetic efficacy of inferior alveolar nerve block plus buccal infiltration or periodontal ligament injections with articaine in patients with irreversible pulpitis in the mandibular first molar. Oral Surgery, Oral Medicine, Oral Pathology, Oral Radiology, and Endodontics.

[ref-16] Foster W, Drum M, Reader A, Beck M (2007). Anesthetic efficacy of buccal and lingual infiltrations of lidocaine following an inferior alveolar nerve block in mandibular posterior teeth. Anesthesia Progress.

[ref-17] George GSt, Morgan A, Meechan J, Moles DR, Needleman I, Ng YL, Petrie A (2018). Injectable local anaesthetic agents for dental anaesthesia. The Cochrane Database of Systematic Reviews.

[ref-18] Gupta A, Wadhwa J, Aggarwal V, Mehta N, Abraham D, Aneja K, Singh A (2022). Anesthetic efficacy of supplemental intraligamentary injection in human mandibular teeth with irreversible pulpitis: a systematic review and meta-analysis. Journal of Dental Anesthesia and Pain Medicine.

[ref-19] Haas DA, Harper DG, Saso MA, Young ER (1991). Lack of differential effect by Ultracaine (articaine) and Citanest (prilocaine) in infiltration anaesthesia. Journal (Canadian Dental Association).

[ref-20] Helmy RH, Zeitoun SI, El-Habashy LM (2022). Computer-controlled Intraligamentary local anaesthesia in extraction of mandibular primary molars: randomised controlled clinical trial. BMC Oral Health.

[ref-21] Higgins JPT, Thomas J, Chandler J, Cumpston M, Li T, Page MJ, Welch VA (2022). Cochrane handbook for systematic reviews of interventions version 6.2 (updated 2022): Cochrane, 2022. www.training.cochrane.org/handbook.

[ref-22] Hjermstad MJ, Fayers PM, Haugen DF, Caraceni A, Hanks GW, Loge JH, Fainsinger R, Aass N, Kaasa S (2011). Studies comparing numerical rating scales, verbal rating scales, and visual analogue scales for assessment of pain intensity in adults: a systematic literature review. Journal of Pain and Symptom Management.

[ref-23] Jain M, Nazar N (2018). Comparative evaluation of the efficacy of intraligamentary and supraperiosteal injections in the extraction of maxillary teeth: a randomized controlled clinical trial. The Journal of Contemporary Dental Practice.

[ref-24] Jensen MP, Karoly P, Braver S (1986). The measurement of clinical pain intensity: a comparison of six methods. Pain.

[ref-25] Kaufman E, Epstein JB, Naveh E, Gorsky M, Gross A, Cohen G (2005). A survey of pain, pressure, and discomfort induced by commonly used oral local anesthesia injections. Anesthesia Progress.

[ref-26] Kennedy M, Reader A, Beck M, Weaver J (2001). Anesthetic efficacy of ropivacaine in maxillary anterior infiltration. Oral Surgery, Oral Medicine, Oral Pathology, Oral Radiology, and Endodontics.

[ref-27] Kliger M, Stahl S, Haddad M, Suzan E, Adler R, Eisenberg E (2015). Measuring the intensity of chronic pain: are the visual analogue scale and the verbal rating scale interchangeable?. Pain Practice.

[ref-28] Lara-Muñoz C, De Leon SP, Feinstein AR, Puente A, Wells CK (2004). Comparison of three rating scales for measuring subjective phenomena in clinical research. I. Use of experimentally controlled auditory stimuli. Archives of Medical Research.

[ref-29] Lasemi E, Sezavar M, Habibi L, Hemmat S, Sarkarat F, Nematollahi Z (2015). Articaine (4%) with epinephrine (1:100, 000 or 1:200, 000) in inferior alveolar nerve block: effects on the vital signs and onset, and duration of anesthesia. Journal of Dental Anesthesia and Pain Medicine.

[ref-30] Liau FL, Kok SH, Lee JJ, Kuo RC, Hwang CR, Yang PJ, Lin CP, Kuo YS, Chang HH (2008). Cardiovascular influence of dental anxiety during local anesthesia for tooth extraction. Oral Surgery, Oral Medicine, Oral Pathology, Oral Radiology, and Endodontics.

[ref-31] Lilienthal B (1975). A clinical appraisal of intraosseous dental anesthesia. Oral Surgery, Oral Medicine, and Oral Pathology.

[ref-32] Malamed SF (1982). The periodontal ligament (PDL) injection: an alternative to inferior alveolar nerve block. Oral Surgery, Oral Medicine, and Oral Pathology.

[ref-33] Malamed SF (1998). Local anesthesia. Journal of the California Dental Association.

[ref-34] Mansour MS, Adawy AM (1985). The periodontal ligament injection; a solitary method for inducing local anesthesia. Egyptian Dental Journal.

[ref-35] Marin MK (1987). Intraseptal anesthesia in the general dental practice. Compendium.

[ref-36] Meechan JG (1992). Intraligamentary anaesthesia. Journal of Dentistry.

[ref-37] Meechan JG, Ledvinka JI (2002). Pulpal anaesthesia for mandibular central incisor teeth: a comparison of infiltration and intraligamentary injections. International Endodontic Journal.

[ref-38] Milgrom P, Coldwell SE, Getz T, Weinstein P, Ramsay DS (1997). Four dimensions of fear of dental injections. Journal of the American Dental Association.

[ref-39] Miller AG (1983). A clinical evaluation of the Ligmaject periodontal ligament injection syringe. Dental Update.

[ref-40] Moore RA, Straube S, Aldington D (2013). Pain measures and cut-offs—‘no worse than mild pain’ as a simple, universal outcome. Anaesthesia.

[ref-41] Nusstein JM, Reader A, Drum M (2010). Local anesthesia strategies for the patient with a hot tooth. Dental Clinics of North America.

[ref-42] O’Rourke D (2004). The measurement of pain in infants, children, and adolescents: from policy to practice. Physical Therapy.

[ref-43] Ram D, Peretz B (2003). The assessment of pain sensation during local anesthesia using a computerized local anesthesia (Wand) and a conventional syringe. Journal of Dentistry for Children.

[ref-44] Reed MD, Van Nostran W (2014). Assessing pain intensity with the visual analog scale: a plea for uniformity. Journal of Clinical Pharmacology.

[ref-45] Ryalat ST, Al-Shayyab MH, Amin W, AlRyalat SA, Al-Ryalat N, Sawair F (2018). Efficacy of intraligamentary anesthesia in maxillary first molar extraction. Journal of Pain Research.

[ref-46] Shabazfar N, Daubländer M, Al-Nawas B, Kämmerer PW (2014). Periodontal intraligament injection as alternative to inferior alveolar nerve block—meta-analysis of the literature from 1979 to 2012. Clinical Oral Investigations.

[ref-47] Shamseer L, Moher D, Clarke M, Ghersi D, Liberati A, Petticrew M, Shekelle P, Stewart LA (2015). Preferred reporting items for systematic review and meta-analysis protocols (PRISMA-P) 2015: elaboration and explanation. BMJ.

[ref-48] Spuller RL (1988). Use of the periodontal ligament injection in dental care of the patient with hemophilia—a clinical evaluation. Special Care in Dentistry.

[ref-49] Subramaniam K, Dhanraj M, Jain AR (2018). Effectiveness of intraligamental anesthesia in controlling the hyperesthesia during tooth preparation. Drug Invention Today.

[ref-50] Williams ACD, Davies HTO, Chadury Y (2000). Simple pain rating scales hide complex idiosyncratic meanings. Pain.

[ref-51] Williamson A, Hoggart B (2005). Pain: a review of three commonly used pain rating scales. Journal of Clinical Nursing.

[ref-52] Wong DL, Baker CM (1988). Pain in children: comparison of assessment scales. Pediatric Nursing.

[ref-53] Woodmansey K (2005). Intraseptal anesthesia: a review of a relevant injection technique. General Dentistry.

[ref-54] Wright EF (2011). Medial pterygoid trismus (myospasm) following inferior alveolar nerve block: case report and literature review. General Dentistry.

[ref-55] Yılmaz E, Çağırır Dindaroğlu F (2023). Comparison of the effectiveness of intraligamentary anesthesia and inferior alveolar nerve block on mandibular molar teeth in pediatric patients: a randomized controlled clinical study. Clinical Oral Investigations.

[ref-56] Younger J, McCue R, Mackey S (2009). Pain outcomes: a brief review of instruments and techniques. Current Pain and Headache Reports.

[ref-57] Youssef BR, Söhnel A, Welk A, Abudrya MH, Baider M, Alkilzy M, Splieth C (2021). RCT on the effectiveness of the intraligamentary anesthesia and inferior alveolar nerve block on pain during dental treatment. Clinical Oral Investigations.

