# Supplementary material for: Comparison of dental anesthetic efficacy between the periodontal intraligamentary anesthesia and other infiltration anesthesia: a systematic review and meta-analysis
Source: PeerJ. 2023 Jul 24;11:e15734. doi: 10.7717/peerj.15734 (PMC10373649; doi:10.7717/peerj.15734)
Supplement: Supplemental Information 2 [file peerj-11-15734-s002.docx]

# Systematic Review and Meta-Analysis Rationale

Up to now, infiltration anesthesia and nerve block anesthesia have been widely used to control pain during dental procedures. However, different anesthesia techniques exhibit different characteristics, which impact their scopes of use (Kaufman et al. 2005).

Intraligamentary anesthesia, as one of the infiltration anesthesia techniques, is often used as supplementary anesthesia. The comparison of pain scores and success rates between intraligamentary anesthesia and other infiltration anesthesia remains controversial. Some literature indicated either higher or lower pain scores during the administration of intraligamentary anesthetic injection than other local infiltration procedures, while other researches showed no significant difference between these techniques in pain scores (Fan et al. 2009; Ram & Peretz 2003).

This is the first systematic review and meta-analysis to compare dental anesthetic effectiveness between periodontal intraligamentary anesthesia and other infiltration anesthesia. According to our results, those anesthesia techniques showed the same success rates, which indicated that intraligamentary anesthesia had the potential as a conventional injection technique. However, intraligamentary anesthesia brought more pain during injection than other infiltration anesthesia but less adverse reactions. In this manuscript, we provided relevant information and suggestions for both clinicians and researchers.

# REFERENCES

**Fan S, Chen WL, Pan CB, Huang ZQ, Xian MQ, Yang ZH, Dias-Ribeiro E, Liang YC, Jiao JY, Ye YS, and Wen TY. 2009**. Anesthetic efficacy of inferior alveolar nerve block plus buccal infiltration or periodontal ligament injections with articaine in patients with irreversible pulpitis in the mandibular first molar. *Oral Surg Oral Med Oral Pathol Oral Radiol Endod* **108**:e89-93. 10.1016/j.tripleo.2009.06.012

**Kaufman E, Epstein JB, Naveh E, Gorsky M, Gross A, and Cohen G. 2005**. A survey of pain, pressure, and discomfort induced by commonly used oral local anesthesia injections. *Anesth Prog* **52**:122-127. 10.2344/0003-3006(2005)52[122:Asp]2.0.Co;2

**Ram D, and Peretz B. 2003**. The assessment of pain sensation during local anesthesia using a computerized local anesthesia (Wand) and a conventional syringe. *J Dent Child (Chic)* **70**:130-133.
