# Supplementary material for: Comparison of dental anesthetic efficacy between the periodontal intraligamentary anesthesia and other infiltration anesthesia: a systematic review and meta-analysis
Source: PeerJ. 2023 Jul 24;11:e15734. doi: 10.7717/peerj.15734 (PMC10373649; doi:10.7717/peerj.15734)
Supplement: Supplemental Information 3 [file peerj-11-15734-s003.docx]

**Table S1. Excluded studies with reasons**

| **Study, Year** | **Reasons for excluding** |
| --- | --- |
| Aggarwal et al. 2009 | supplemental buccal-plus-lingual infiltration after IANB |
| Aggarwal et al. 2010 | compare the efficacy of IANB with buccal-plus-lingual infiltration |
| Arali et al. 2015 | compare the efficacy of IANB with BI |
| Chompu-Inwai et al. 2018 | supplemental intraligamentary anesthesia after IANB |
| Crump et al. 2022 | supplemental intraligamentary anesthesia for palatal infiltration |
| Dianat et al. 2020 | compare the efficacy of IANB with IANB plus BI |
| Dou et al. 2013 | supplemental BI and buccal-plus-lingual infiltration after IANB |
| Dumbrigue et al. 1997 | compare the efficacy of IANB with intraligamentary anesthesia |
| Fan et al. 2009 | compare the efficacy of IANB plus BI with IANB plus intraligamentary anesthesia |
| Helmy et al. 2022 | compare the efficacy of IANB with CC-ILA |
| Kämmerer et al. 2018 | compare the efficacy of IANB with intraligamentary anesthesia |
| Kanaa et al. 2009 | compare the efficacy of IANB with IANB plus BI |
| Matthews et al. 2009 | supplemental infiltration anesthesia after IANB |
| Oulis 1996 | compare the efficacy of IANB with infiltration anesthesia |
| Parirokh et al. 2014 | compare the efficacy of IANB with IANB + BI + intraligamentary anesthesia |
| Runnacles et al. 2015 | no crucial data |
| Saber et al. 2022 | compare the efficacy among IANB, IANB plus BI and IANB plus infiltration anesthesia |
| Singhal et al. 2022 | supplemental BI and intraligamentary anesthesia after IANB |
| Tekin et al. 2012 | compare the efficacy of IANB with intraligamentary anesthesia |
| Thiem et al. 2018 | compare the efficacy of IANB with infiltration anesthesia |
| Yilmaz et al. 2018 | compare the efficacy of IANB with BI |
| Yılmaz et al. 2023 | compare the efficacy of IANB with intraligamentary anesthesia |
| Youssef et al. 2021 | compare the efficacy of IANB with intraligamentary anesthesia |
| Zargar et al. 2022 | supplemental BI and intraligamentary anesthesia after IANB |

IANB: inferior alveolar nerve block; BI: buccal infiltration; CC-ILA: Computer-controlled Intraligamentary anesthesia (CC-ILA)

# REFERENCE

**Aggarwal V, Jain A, Kabi D. 2009**. Anesthetic Efficacy of Supplemental Buccal and Lingual Infiltrations of Articaine and Lidocaine after an Inferior Alveolar Nerve Block in Patients with Irreversible Pulpitis. *Journal of endodontics* **35**:925-929. DOI 10.1016/j.joen.2009.04.012

**Aggarwal V, Singla M, Kabi D. 2010**. Comparative evaluation of anesthetic efficacy of Gow-Gates mandibular conduction anesthesia, Vazirani-Akinosi technique, buccal-plus-lingual infiltrations, and conventional inferior alveolar nerve anesthesia in patients with irreversible pulpitis. *Oral Surgery, Oral Medicine, Oral Pathology, Oral Radiology and Endodontology* **109**:303-308. DOI 10.1016/j.tripleo.2009.09.016

**Arali V, Mytri P. 2015**. Anaesthetic efficacy of 4% articaine mandibular buccal infiltration compared to 2% lignocaine inferior alveolar nerve block in children with irreversible pulpitis. *Journal of Clinical and Diagnostic Research* **9**:ZC65-ZC67. DOI 10.7860/JCDR/2015/12821.5856

**Chompu-Inwai P, Sutharaphan T, Nirunsittirat A, Chuveera P, Srisuwan T, Sastraruji T. 2018**. How effective are inferior alveolar nerve block and supplemental intraligamentary injections in pediatric patients with deep carious permanent mandibular molars? *Pediatric dentistry* **40**:437-442.

**Crump B, Reader A, Nusstein J, Drum M, Fowler S, Draper J. 2022**. Prospective Study on PDL Anesthesia as an Aide to Decrease Palatal Infiltration Pain. *Anesthesia progress* **69**:10-17. DOI 10.2344/anpr-68-03-03

**Dianat O, Mozayeni MA, Layeghnejad MK, Shojaeian S. 2020**. The efficacy of supplemental intraseptal and buccal infiltration anesthesia in mandibular molars of patients with symptomatic irreversible pulpitis. *Clinical oral investigations* **24**:1281-1286. DOI 10.1007/s00784-019-03006-8

**Dou L, Luo J, Yang D. 2013**. Anaesthetic efficacy of supplemental lingual infiltration of mandibular molars after inferior alveolar nerve block plus buccal infiltration in patients with irreversible pulpitis. *International endodontic journal* **46**:660-665. DOI 10.1111/iej.12042

**Dumbrigue HB, Lim MVC, Rudman RA, Serraon A. 1997**. A comparative study of anesthetic techniques for mandibular dental extraction. *American Journal of Dentistry* **10**:275-278.

**Fan S, Chen WL, Pan CB, Huang ZQ, Xian MQ, Yang ZH, Dias-Ribeiro E, Liang YC, Jiao JY, Ye YS, Wen TY. 2009**. Anesthetic efficacy of inferior alveolar nerve block plus buccal infiltration or periodontal ligament injections with articaine in patients with irreversible pulpitis in the mandibular first molar. *Oral surgery, oral medicine, oral pathology, oral radiology, and endodontics* **108**:e89-93. DOI 10.1016/j.tripleo.2009.06.012

**Helmy RH, Zeitoun SI, El-Habashy LM. 2022**. Computer-controlled Intraligamentary local anaesthesia in extraction of mandibular primary molars: randomised controlled clinical trial. *BMC Oral Health* **22**. DOI 10.1186/s12903-022-02194-2

**Kämmerer PW, Adubae A, Buttchereit I, Thiem DGE, Daubländer M, Frerich B. 2018**. Prospective clinical study comparing intraligamentary anesthesia and inferior alveolar nerve block for extraction of posterior mandibular teeth. *Clinical oral investigations* **22**:1469-1475. DOI 10.1007/s00784-017-2248-2

**Kanaa MD, Whitworth JM, Corbett IP, Meechan JG. 2009**. Articaine buccal infiltration enhances the effectiveness of lidocaine inferior alveolar nerve block. *International endodontic journal* **42**:238-246. DOI 10.1111/j.1365-2591.2008.01507.x

**Matthews R, Drum M, Reader A, Nusstein J, Beck M. 2009**. Articaine for Supplemental Buccal Mandibular Infiltration Anesthesia in Patients with Irreversible Pulpitis When the Inferior Alveolar Nerve Block Fails. *Journal of endodontics* **35**:343-346. DOI 10.1016/j.joen.2008.11.025

**Oulis CJ. 1996**. The effectiveness of mandibular infiltration compared to mandibular block anesthesia in treating primary molars in children. *Pediatric dentistry* **18**:301-305.

**Parirokh M, Sadr S, Nakhaee N, Abbott PV, Askarifard S. 2014**. Efficacy of supplementary buccal infiltrations and intraligamentary injections to inferior alveolar nerve blocks in mandibular first molars with asymptomatic irreversible pulpitis: a randomized controlled trial. *International endodontic journal* **47**:926-933. DOI 10.1111/iej.12236

**Runnacles P, Arrais CA, Pochapski MT, dos Santos FA, Coelho U, Gomes JC, De Goes MF, Gomes OM, Rueggeberg FA. 2015**. Direct measurement of time-dependent anesthetized in vivo human pulp temperature. *Dental materials : official publication of the Academy of Dental Materials* **31**:53-59. DOI 10.1016/j.dental.2014.11.013

**Saber SM, Hashem AA, Khalil DM, Pirani C, Ordinola-Zapata R. 2022**. Efficacy of four local anaesthesia protocols for mandibular first molars with symptomatic irreversible pulpitis: A randomized clinical trial. *International endodontic journal* **55**:219-230. DOI 10.1111/iej.13667

**Singhal N, Vats A, Khetarpal A, Ahlawat M, Vijayran VKR, Harshita. 2022**. Efficacy of articaine versus mepivacaine administered as different supplementary local anesthetic techniques after a failed inferior alveolar nerve block with lidocaine in patients with irreversible pulpitis: An in vivo study. *Journal of conservative dentistry : JCD* **25**:654-660. DOI 10.4103/jcd.jcd_299_22

**Tekin U, Ersin N, Oncag O, Bent B, Menderes M, Kocanali B. 2012**. Comparison of inferior alveolar nerve block and intraligamentary anesthesia on the discomfort of children. *Journal of International Dental and Medical Research* **5**:143-148.

**Thiem DGE, Schnaith F, Van Aken CME, Köntges A, Kumar VV, Al-Nawas B, Kämmerer PW. 2018**. Extraction of mandibular premolars and molars: comparison between local infiltration via pressure syringe and inferior alveolar nerve block anesthesia. *Clinical oral investigations* **22**:1523-1530. DOI 10.1007/s00784-017-2251-7

**Yılmaz E, Çağırır Dindaroğlu F. 2023**. Comparison of the effectiveness of intraligamentary anesthesia and inferior alveolar nerve block on mandibular molar teeth in pediatric patients: a randomized controlled clinical study. *Clinical oral investigations*. DOI 10.1007/s00784-023-04911-9

**Yilmaz K, Tunga U, Ozyurek T. 2018**. Buccal infiltration versus inferior alveolar nerve block in mandibular 2nd premolars with irreversible pulpitis. *Nigerian Journal of Clinical Practice* **21**:473-477. DOI 10.4103/njcp.njcp_135_17

**Youssef BR, Söhnel A, Welk A, Abudrya MH, Baider M, Alkilzy M, Splieth C. 2021**. RCT on the effectiveness of the intraligamentary anesthesia and inferior alveolar nerve block on pain during dental treatment. *Clinical oral investigations* **25**:4825-4832. DOI 10.1007/s00784-021-03787-x

**Zargar N, Shojaeian S, Vatankhah M, Heidaryan S, Ashraf H, Akbarzadeh Baghban A, Dianat O. 2022**. Anesthetic efficacy of supplemental buccal infiltration versus intraligamentary injection in mandibular first and second molars with irreversible pulpitis: a prospective randomized clinical trial. *Journal of dental anesthesia and pain medicine* **22**:339-348. DOI 10.17245/jdapm.2022.22.5.339
